# The microbiome of the human lower airways: a next generation sequencing perspective

**DOI:** 10.1186/s40413-015-0074-z

**Published:** 2015-06-16

**Authors:** Velma T. E. Aho, Pedro A. B. Pereira, Tari Haahtela, Ruby Pawankar, Petri Auvinen, Kaisa Koskinen

**Affiliations:** 1 DNA Sequencing and Genomics Laboratory, Institute of Biotechnology, University of Helsinki, P.O. Box 56 (Viikinkaari 4), 00014 Helsinki, Finland; 2 Skin and Allergy Hospital, Helsinki University Hospital, Helsinki, Finland; 3 Division of Allergy, Department of Pediatrics, Nippon Medical School, Tokyo, Japan; 4 Faculty of Veterinary Medicine, University of Helsinki, Helsinki, Finland

## Abstract

For a long time, the human lower airways were considered a sterile environment where the presence of microorganisms, typically revealed by culturing, was interpreted as an abnormal health state. More recently, high-throughput sequencing-based studies have led to a shift in this perception towards the notion that even in healthy conditions the lower airways show either transient presence or even permanent colonization by microorganisms. However, challenges related to low biomass and contamination in samples still remain, and the composition, structure and dynamics of such putative microbial communities are unclear. Here, we review the evidence for the presence of microbial communities in the human lower airways, in healthy subjects and within the context of medical conditions of interest. We also provide an overview of the methodology pertinent to high-throughput sequencing studies, specifically those based on amplicon sequencing, including a discussion of good practices and common pitfalls.

## Introduction

The human body harbours a varied, complex, and dynamic community of microorganisms known as the human microbiome. The term “microbiome” was originally coined by Joshua Lederberg [1]. Lederberg was specifically referring to the *human* microbiome, but since then the term has been extended to other environments as well. The original definition does not explicitly state whether the physicochemical characteristics of the environment in question are to be included, although not including them would make “microbiome” synonymous with “microbiota”. We prefer to make an explicit distinction that takes into account the ecological concept of “biome”. Therefore, the human microbiome consists of bacteria, archaea, protists, fungi, their respective viruses, and human viruses, as well as the surrounding host environment in which they exist. In practice, the terms are often used interchangeably, including in the present paper, but the meaning is nevertheless readily understood from context.

It has been estimated that the number of bacterial cells in the human body may exceed our own cells by an order of magnitude ([2], but see discussion in [3]). Some of these resident organisms live in a state of commensalism with their human host, making out a living from the refuse of the host and/or from the metabolic by-products of other microbes, while others are part of a mutualistic relationship, in which both host and microbe benefit. Opportunistic pathogens are microorganisms that are commonly present in healthy individuals in low levels and that may become problematic due to abnormal outgrowth promoted by microbial community disruption and/or due to a compromised immune system.

Culture-independent methods have enabled the detection of bacteria in some rather unexpected niches in the human body. The lower respiratory tract of healthy individuals has been considered a sterile environment where the presence of any bacteria, typically revealed by culturing, represents an abnormal, unhealthy state [4, 5]. As with many other environments, this may have been caused by the fact that the microbes present are difficult to culture under standard laboratory conditions. Recently, numerous studies, particularly those based on high-throughput sequencing of the gene coding for the 16S ribosomal RNA, have provided tantalizing evidence that bacteria may be present in the lower respiratory tract even in healthy subjects. There are challenges in such studies, particularly related to the low biomass of the starting material and contamination of the samples [6, 7]. Nevertheless, a growing number of studies are documenting the microbiome in healthy airways and in relation to medical conditions such as cystic fibrosis [8, 9], chronic obstructive pulmonary disease [10], asthma [11] and lung transplantation [12]. In some medical conditions, the sequencing data is supported by evidence from culturing methods [13, 14], but many studies rely entirely on a culture-independent approach.

Here, we provide an overview of DNA sequencing-based methodology and the microbiome of the human lower airways, mainly focusing on bacteria, from a 16S rRNA marker gene high-throughput amplicon sequencing perspective. The reasons for this taxonomic and technical focus are mostly practical, and include: 1) the vast majority of published studies deal specifically with bacteria; 2) phylogenetic marker gene studies that take into account other taxa require their own specific primers and downstream processing, increasing the cost and duration of the studies; 3) lack of standardized protocols and easy implementation and analysis; 4) less is known about other taxonomic groups within a human microbiome context, leading to weaker theoretical evidence from which to derive mechanistic hypotheses regarding relationships with the human host; 5) many of those groups have somewhat less relevance to human disease when compared to bacteria; and 6) shotgun metagenomics is significantly more expensive and requires much more complex and time-consuming analysis.

## A brief overview of methods in microbiome studies

### Next generation sequencing in microbial ecology

Next generation sequencing technologies have been widely applied in microbial ecology research. The most utilized approaches include shotgun metagenomics and amplicon sequencing. The term metagenomics was first used by Handelsman and colleagues [15], and it denotes examining the structure and function of the entire (microbial) community based on data derived from bulk genetic material extracted directly from samples. Therefore, the produced sequence data contains a fragmented puzzle of genomes of bacteria, archaea, viruses, fungi, protists, and even plants and animals, depending on the studied environment [16].

The first large-scale metagenomics study was conducted by Venter and colleagues in the Sargasso Sea [17]. They showed that by applying whole-genome shotgun sequencing to microbial populations it is possible to assess the diversity and community structure, and to discover and at least partially reconstruct previously unknown microbial genomes. Their results showed that a considerable share of the detected bacteria were indeed unfamiliar and resistant to cultivation by common laboratory methods, and therefore emphasized the importance of DNA-based techniques in studying diverse and still largely unknown microbial populations.

Amplicon sequencing can be defined as a targeted version of metagenomics: a specific genetic region shared by the community members of interest is amplified using universal primers. The produced fragments of similar length are then sequenced. Amplification success is assumed equal across taxa, and hence each sequence read randomly represents the genetic diversity in the studied sample. Typically, the amplified fragment is part of a phylogenetically or functionally informative gene, such as the 16S ribosomal RNA gene or other marker genes [18–20]. Therefore, 16S rRNA amplicon sequencing is, strictly speaking, not metagenomics, although it is common to describe it as such. The choice of which variable region or regions of the 16S rRNA gene to target for sequencing is not trivial, since it affects what taxa are recovered and how accurately they can be classified [21, 22].

**Fig. 1:**
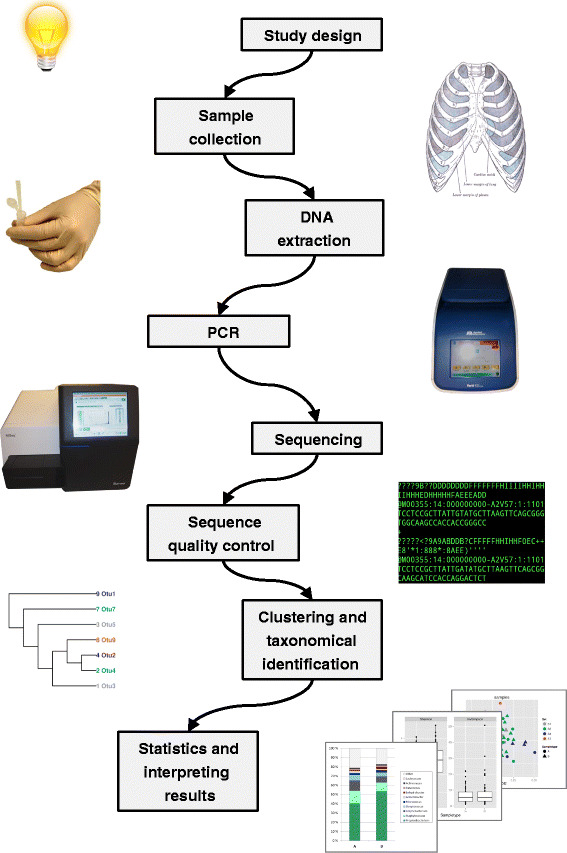
General workflow of a microbiome study, from design to data analysis. This schematic is specific for target gene sequencing-based studies (e.g. 16S rRNA gene) and is not representative of studies using other approaches, e.g. shotgun metagenomics studies

Human microbiome studies are a branch of microbial ecology. The goal is to study the variation of microbial communities in regard to, for example, population, genotype, age, nutrition, medication, biotic and abiotic environment, and/or its influence on disease. Figure 1 summarizes the typical workflow of an amplicon-based microbiome study. The first step in any such project is study design, where many details such as selection criteria for subjects, type of samples collected, and the methods used in laboratory and data analysis must be considered. Sample collection has its specific challenges, such as avoiding contamination. Once the samples have been obtained, the laboratory workflow includes DNA extraction, PCR amplification, purification, and sequencing. For sequencing, there are several different platforms, each with their pros and cons. The resulting sequence data is analysed bioinformatically, and finally, compared with statistical tools. All these steps and their potential obstacles will be briefly reviewed below. For further discussion on conducting a microbiome study, see [23].

### Sampling and sample processing

Sampling of the lower airways is typically based on bronchoalveolar lavage (BAL) or collection of induced sputum (Table 1). Contamination of samples taken from low biomass environments is a pervasive problem in DNA sequence-based studies [7]. It can occur during sample collection and/or during sample processing at the pre-sequencing stage. Completely aseptic methods of sample collection, storage, and processing are a practical impossibility, but the risk of contamination-derived problems is exacerbated when the biomass of the targeted microbiota is low [24]. This is often the case in human microbiome studies that rely on samples where few microbes are present, such as skin swabs or BAL fluid. The lower the microbial biomass of the sample, the greater the possibility that due to low concentration or quality of the obtained DNA, contaminants will swamp the real signal being sought. Potential sources of contamination include swabs, biopsy needles, collector, storage and reaction vessels, carryover by bronchoscopes, as well as the individual performing the sampling, either by accident or mishandling. To our knowledge, no thorough, systematic studies have been conducted to evaluate the potential for contamination in these particular cases.

**Table 1:** Next generation 16S rRNA gene amplicon based sequencing studies of the lower airway microbiome

Reference^a^	Medical condition	n (cases + controls)	Lower respiratory tract sample type	Sequencing platform	Targeted 16S rRNA regions	Sequence analysis software^b^
[108]	arsenic exposure	10 + 10	sputum	IonTorrent	V6	QIIME
[91]	asthma	10 + 10	sputum	454	V6	RDP
[92]	asthma	39 + 12	BAL	454	V1V2	BARTAB, RDP
[56]	CF	23	sputum	454	V1V2	AbundantOTU, Lucy, mothur, RDP
[59]	CF	10	lung explant secretions, sputum	454	V1V3, V2V3	KrakenBLAST, QIIME
[57]	CF	17	sputum	454	V4V6	custom in-house pipeline
[60]	CF	25	sputum	454	V1V3	RDP
[58]	CF	23	sputum	454	V6V7	QIIME
[109]	CF	19 + 6	sputum, lung tissue	454	V4V5	Matlab, mothur, RDP
[110]	CF	21	sputum	454	V1V2	BARTAB, RDP
[111]	CF	30	sputum	454	V1V3	custom software, Kraken BLAST
[68]	COPD	11	sputum	MiSeq	V4	QIIME
[42]	COPD, smoking	19 + 3	BAL, lung tissue	454	V1V3	mothur, RDP
[66]	COPD, smoking	16 + 16	lung tissue	454	V1V3	mothur
[41]	healthy	6	BAL, lower airway protected brush	454	V1V2	QIIME
[49]	healthy	28	BAL	454	V3V5	mothur
[44]	healthy smokers	19 + 45	BAL	454	V1V3, V3V5	mothur
[52]	HIV	82 + 77	BAL	454	V1V3	QIIME
[107]	ILD	24 + 9	BAL	454	V3V5	PyroTagger
[6]	intubation	5	ETA	454	V1V3	MG-RAST, mothur
[104]	IPF	55	BAL	454	V3V5	mothur
[105]	IPF	65 + 44	BAL	454	V3V5	QIIME
[96]	lung transplantation	4 + 2	BAL	454	V3	mothur, RDP
[95]	lung transplantation	21	BAL	454	V1V2	QIIME
[12]	lung transplantation	33 + 26	BAL	454	V3V5	mothur
[97]	lung transplantation	57 + 8	BAL	454	V7V8	QIIME
[102]	non-CF bronchiectasis	41	BAL, sputum	454	V1V3	custom software, Kraken BLAST
[103]	non-CF bronchiectasis	42	sputum	454	V1V3	custom software, Kraken BLAST
[101]	non-CF bronchiectasis	40	sputum	454	V1V3	AbundantOTU, Lucy, mothur, RDP
[45]	smoking, pulmonary inflammation	20 + 9	BAL	454	V1V2	QIIME
[106]	tuberculosis	22 + 14	sputum	454	V1V2	QIIME
[43]	various	6	BAL	454	V1V2	QIIME
[112]	various	56 + 4	BAL, sputum	454	V1V3	mothur

^a^In the case of several publications using the same sequence data, only the first one is included

^b^Software for diversity calculation, statistical comparisons etc. is not included. RDP refers to the standalone implementation. If RDP was used via mothur or QIIME, it is not listed

Of special interest are the data on number of cases and controls, sample type, and 16S rRNA gene regions targeted for sequencing

Contamination issues are not confined to sample collection; the problem is present in sample preparation at all stages. Different DNA extraction kits have been shown to contain different bacterial taxa as contaminants ([7], unpublished personal observations). Even different lots from the same kit can show variability in taxa and amount of contaminant biomass. Yet another potential source of contamination are the laboratory reagents, such as enzymes, lab-grade water, and buffers. Additionally, the problem can be exacerbated during PCR amplification itself, in which with increasing PCR cycle number there is a corresponding increase in the production of contaminant sequences. Although the amount of the targeted amplicons will also increase and under ideal circumstances the proportions between both should be maintained, there may be biased amplification of the undesirable sequences depending on the primer sequences and DNA sequencing technology used (unpublished personal observations). This is not a significant issue with high biomass samples (e.g. stool), because the starting biomass is so high that there is no need for a large number of PCR cycles, and therefore the potential contaminant DNA will be suppressed by the real signal.

There are various approaches that can be used to minimize the effects of contamination and that are easy to implement in most lab settings. Table 2 summarises some of those approaches, as well as other general considerations that are important for effective sample handling and processing.

**Table 2:** Good practices in microbiome studies

Design considerations	• The bacterial “universal” primers show bias in PCR amplification of certain taxa, and the use of different regions of the 16S rRNA gene in different studies (as seen for the lower airways in Table 1) affects inter-study comparisons (e.g. [21, 22])
Sample collection	• Make sure that collecting and storage vessels are not needlessly subjected to potential contamination either by body contact or air exposure.
DNA extraction	• Perform all activities (as long as it is practical to do so) under a hood with air filtering.
	• Samples should be randomized before DNA extraction so that batch effects are minimized when groups of interest (case vs. control, age, sex, treatment, etc.) are compared. Varying contamination in kit lots and laboratory reagents can create artificial differences between groups during statistical analysis if the samples are not handled in randomized fashion (see [7] for an excellent and clear example).
	• Be generous with controls. Every batch of samples being isolated at the same time should include one kit “blank” (control) to which no sample material is added but which undergoes the same process of DNA extraction and sequencing as the “real” samples. This serves the purpose of controlling for both contamination present in the kit and contamination introduced during the extraction process.
	• Keep records of which kit lot was used for DNA extraction of which sample, and don’t mix reagents from different kit boxes, even if from the same lot.
	• Use kits with bead-beating to increase the chances that taxa with thicker cell walls will be properly lysed and that taxonomic representation biases will be avoided as much as possible.
	• Ensure that all samples from the same project are handled in the same way following a common protocol, each individual step preferably executed by the same person.
PCR	• When possible, work in a PCR clean room.
	• Sequence a PCR master mix “blank” (control) for each different master mix aliquot. Do not add any template. Master mix controls serve the purpose of detecting potential contamination in PCR reagents (already present or accidentally introduced during preparation) every time a new master mix is prepared.
	• Use PCR replicates to minimize PCR bias (uneven PCR amplification, lack of reaction effectiveness) and to detect the diversity present in samples as thoroughly as possible [113]. A minimum of two replicate reactions should be prepared per sample.
Sequencing	• If possible, sequence a mock community prepared from genomic DNA from known isolates. Since the sequence composition of this community is known, it can be used to identify contamination effects and sequencing errors in the target samples.

This list is provided as an example of technical considerations that must be taken into account in studies involving DNA sequencing of environmental samples. The second and third sections are important for sequence-based studies in general, while the first, fourth, and fifth sections are of special interest for studies requiring target DNA amplification prior to sequencing

There have been suggestions for treating reagents from extraction kits and PCR mixes using, for example, different types of radiation, restriction enzymes, or DNases [7]. All of these decontamination procedures have advantages and disadvantages, and their use needs to be evaluated within the context of the study in mind. Additionally, sequencing the complete genomes of common contaminants to identify and computationally subtract them from the data, will in the future help to distinguish between contaminants and real signal [24]. For a comprehensive review of these issues, as well as suggestions for dealing with them, see [7] and references therein.

### Sequencing, bioinformatics and data analysis

The majority of published 16S rRNA gene sequencing based lower airway microbiome studies have used Roche’s 454 pyrosequencing platform (Table 1). With the forthcoming withdrawal of 454 from the market, Illumina’s sequencers are quickly becoming the standard for both amplicon and shotgun metagenomics studies. The Illumina MiSeq platform in particular will probably eventually dominate amplicon-based microbial surveys. The various existing sequencing platforms have marked differences in read lengths, throughput, run time, error profiles, and cost [25, 26].

Regardless of the sequencing platform used, the raw sequence data requires several quality control steps to avoid bias and artefacts caused by the PCR amplification and sequencing [27] Low quality reads are removed based on criteria such as quality scores, mismatches to barcodes and primers, sequence length, and the presence of ambiguous bases. Chimeric reads, which are the result of PCR products where two or more different template sequences have accidentally been joined together, are also removed [27]. After quality control, the sequences are typically clustered to operational taxonomic units (OTUs). Clustering can be performed based on only the sequences themselves (“de novo”), using a reference database of known sequences as cluster centroids (“closed-reference OTU picking”), or with a combination of the two approaches (“open-reference OTU picking”) [28]. Regardless of the method, clustering is usually based on a 3% dissimilarity cut-off, which is used as a proxy for species [29]. It is also possible to omit the clustering step entirely and to simply use a reference database to identify each sequence read, binning together those that have the same taxonomic classification (“phylotype” approach). All methods that rely on a reference database are highly dependent on the quality of the database, while the database-independent “de novo” approach is computationally heavier than the other alternatives. It is particularly important to note what approach has been used when comparing different studies, since different clustering methods can produce widely varying results [30, 31]. After clustering, the resulting OTUs are identified taxonomically using a suitable reference database. This is another step where different databases may lead to major differences in the results.

An additional quality control consideration particularly for studies using low biomass samples is that the data may include contaminants [7]. The data from samples should be compared to sequenced negative controls and to existing publications, to look for unexpected discrepancies that could suggest contamination.

There is a large selection of software available for each step in the analysis of 16S rRNA gene sequence data, from quality control to clustering and taxonomical classification. Instead of using a different tool for each step, many researchers prefer software packages that cover the complete workflow from raw sequence data to final results. The most commonly used ones are mothur [32] and QIIME [33], and the choice between the two is more of a question of personal preference, since both can be used to perform nearly the same tasks. The bioinformatic workflow should always be documented as meticulously as the laboratory work, to ascertain that it is replicable.

The results of the bioinformatic sequence analysis typically include representative sequences for the OTUs and a table that summarizes how many sequences of each OTU are present in each sample. The total amount of sequences per sample can vary significantly, and it is important to take this library size variation into account in statistical comparisons. Traditionally, this has been done by rarefaction or by converting the counts into relative abundances, but neither approach is optimal [34], and there are alternatives, such as the normalization methods offered by the metagenomeSeq [35] and phyloseq [34] R packages. Depending on the study design, the microbial community data can be used for a wide variety of statistical tests, which include, but are not limited to, searching for OTUs that are over or underrepresented in some predefined group (e.g. test subjects with a specific medical condition), comparisons of microbial diversity within and between samples or groups, and multivariate statistics for exploring the possible associations between clinical data and community composition. If OTUs of particular interest are discovered, the sequence data can be used for further analyses of the OTUs in question, both in continued bioinformatic and statistical analysis as well as in future experiments that focus on specific taxa instead of the entire microbiome.

As an end note, by microbial diversity we mean a measurement that takes into account both species richness (number of different taxa) and evenness (how abundant the taxa are). For any given sample, this would be represented by alpha diversity using such indices as Shannon or Inverse Simpson. Beta diversity consists of a distance measure between samples that represents the compositional dissimilarity or heterogeneity between those same samples. Typically, the higher the value of the beta diversity index for any given comparison the higher the dissimilarity between the samples. Notice that different indices have different meanings from an ecological point of view, and their interpretation is not always straightforward. In this review, we try to avoid ambiguity by identifying the meaning of “diversity” as used by the authors of specific articles mentioned in the text. For more information on this topic we suggest the articles by Jost [36, 37], Tuomisto [38], and Anderson et al. [39], and references therein.

### The future

The development of DNA-based methods has already changed our knowledge of the structure and function of our genome as well as our second, microbial genome. Although DNA sequencing is going to become even cheaper in the future, easing the financial constraints on human microbiome studies, many problems remain that are related to study design and analysis of the results rather than just producing data. Bioinformatic tools are still being developed, and comparing results from report to report can be challenging due to the lack of consensus in analysis and presentation of data. Since new methods may require tailor-made analysis, this problem is likely to remain unsolved in the foreseeable future.

Shotgun metagenome projects, which avoid many of the limitations of the 16S rRNA gene approach, are gaining in popularity. They require no selected regions, just brute force sequencing. However, they also add new obstacles to the data analysis, making it more complex and significantly heavier computationally. On the other hand, the results will be more comprehensive and more to the point as reference databases are being filled with well annotated genomes and metagenomes. More functional insight can be obtained, especially as more RNA analyses are performed. Clinical diagnostics will be driven to sequencing-based approaches in the near future. Single-molecule sequencers, such as PacBio and the upcoming nanopore solutions, will be used to rapidly evaluate samples from unknown infections. Mass spectrometry is developing nearly as fast as DNA sequencing, opening novel opportunities in research and diagnostics. Still, sampling is the key to success. When samples are small or of limited quality, contamination problems will remain serious when these very sensitive assays are used.

## The microbiome in healthy lower airways

The first studies using molecular methods to characterize the microbiome of the lower airways concluded that while the lower airways of healthy subjects are not sterile, the amount of bacteria is low, and the community is mainly made up of bacterial genera that are also common in the upper respiratory tract, including *Prevotella, Streptococcus and Veillonella* [40–42]. *Haemophilus* spp. [40] and *Tropheryma whipplei* [41] were suggested as bacteria specific to the lower airways. Later analysis of healthy subjects from an earlier study [41] suggested that three out of six subjects had some OTUs that were enriched in BAL fluid compared to oral wash samples, but all of them were found to be common in either negative controls or peri-glottic samples [43].

In more recent publications, several approaches have been used to eliminate possible contamination and to define statistically which bacteria are typical of the healthy lower respiratory tract. One study listed OTUs classified as *Ralstonia, Bosea, Haemophilus, Methylobacterium, Tropheryma* and an unclassified *Enterobacteriaceae* OTU as potentially lung-specific [44]. Although samples with communities similar to negative controls were removed from the analysis, many of the genera on this list (particularly *Ralstonia, Bosea* and *Methylobacterium,* as well as *Enterobacter,* a genus of *Enterobacteriaceae*) correspond to a published list of common contaminants [7], raising questions regarding their actual origin. Another fairly recent study suggested two “pneumotypes”, one of them including genera similar to “background” or negative control samples, the other with a community resembling that of supraglottic samples, with high levels of *Prevotella* and *Veillonella* [45]*.* The negative controls and the samples similar to them were shown to contain a *Streptococcus* species different from the one typically found in supraglottic samples and supraglottic-like BAL samples. However, it is possible that the background-like “pneumotype” does not represent a true bacterial community, especially since the BAL samples were centrifuged before DNA isolation and only the supernatant was used, which is likely to have an impact on the resulting bacterial communities.

When viewed critically, the existing publications that strive to characterize the microbiome of the healthy lower respiratory tract are not without problems. Many of the bacteria common to the upper airways that have also been detected in the lower airways are likely to originate from microaspiration (subclinical aspiration of small droplets) of oropharyngeal secretions (e.g. [46]), which could mean that their presence is transient. It has been suggested that there is no lung “core” microbiome that is similar across study subjects and stable over time, but that the oral microbiome influences the microbial content in lungs [47]. Another possible source could be gastric reflux [48]. Contamination during sampling may also play a role. On the other hand, recent studies have shown that the BAL microbiome can be sampled without upper respiratory tract contamination [45] and that the lung microbes differ significantly from oral and gastric bacterial community [49]. Additionally, it seems that the sampling route (via nose or mouth) does not significantly affect the BAL microbiome [12].

Finally, it is also important to keep in mind that the standard 16S rRNA gene amplicon sequencing approach does not differentiate living, active cells from dead ones, raising the possibility that, at least in part, the recovered DNA has its origins in non-viable bacteria. Supporting this view is a study in healthy young pigs in which DNase was added to porcine BAL fluid and lung tissue samples [50]. While total bacterial DNA was abundant in both sample types, treatment with DNase substantially reduced the amount of cultivable cells, and showed a total DNA reduction of 63% in BAL samples. On the other hand, Venkataraman et al. [51] showed evidence that 61% of 16S rRNA gene sequences recovered from healthy lung BAL could be matched to species recovered by a diverse assortment of culturing techniques. Of further interest, the study supports a neutral model of community ecology in which most of the bacteria in healthy (as opposed to diseased) lungs are the result of dispersal sourced mainly from the oral cavity, with dispersal and ecological drift dominating over selective forces in shaping healthy lung communities.

Out of the genera suggested to be enriched in the lower airways, some, such as *Haemophilus* [40, 44] and *Tropheryma* [41, 44, 52], have been reported in multiple studies, and are not known contaminants [7]. Further research is needed to ascertain whether there truly is a specific, stable lower airway microbiome in healthy subjects, or if the bacteria that have been discovered from such subjects are only transient visitors [48].

## The lower airway microbiome and specific medical conditions

### Cystic fibrosis

Cystic fibrosis (CF) is a recessive genetic disease particularly common in Caucasian populations [53]. It is caused by mutations that lead to the loss of function or dysfunction of the CF transmembrane conductance regulator protein (CFTR), which among other things causes abnormal mucous secretions and low airway surface pH, facilitating bacterial colonization of the lung [8]. Lung failure associated with chronic airway infections is the most common cause of morbidity and mortality in CF [9]. Based on the amount of publications (Table 1), CF is the lower respiratory tract related condition most commonly studied with next generation 16S rRNA gene sequencing.

CF airway infection is caused by a complex community of microbes [54], but only a handful of bacterial species have been established as clinically relevant. Particularly important pathogens are diverse strains of *Pseudomonas aeruginosa,* and the *Burkholderia cepacia* complex (Bcc) [8]. Other notable species include *Haemophilus influenzae* and *Staphylococcus aureus*, both common in paediatric CF patients, and emergent pathogens such as *Stenotrophomonas maltophilia, Achromobacter xylosoxidans, Mycobacterium abscessus* and the *Streptococcus milleri* group [8, 9]. In addition to recognized pathogens, many genera typical to the upper airways and to healthy lower airways, such as *Prevotella* and *Veillonella,* are also present in CF patients’ lower airways [8, 9]. A study where the taxa found in CF sputum samples were partitioned statistically to satellite and core groups suggested that the core genera include *Porphyromonas, Prevotella, Streptococcus, Catonella, Veillonella, Neisseria* and *Pseudomonas* [55].

Several studies have shown that the microbiome of the CF airways is very stable: neither clinical exacerbation nor antibiotic treatment have a significant effect on the patients’ microbiota [56, 57]. A recent study reported that although antibiotic treatment of *P. aeruginosa* infected CF patients with pulmonary exacerbations initially caused a drop in the relative abundance of *Pseudomonas* and an overall increase in alpha diversity (measured with the Shannon diversity index), the microbial communities reverted to their pre-treatment state within 8–10 days of starting the treatment [58].

A majority of published CF studies have used sputum samples (Table 1), which are thought to represent the lower airway microbiota [59]. One study reports that the communities in sputum and in mouthwash samples are extremely similar, except for a small number of taxa, but suggests that this is probably due to microaspiration, referring to earlier studies as proof that sputum and oral microbiota are different [56]. However, another study using secretions obtained from lung explants concludes that sputum samples are not good surrogates for the lung microbiome, which is mainly dominated by typical CF pathogens and less diverse than the upper airway microbiome [59]. A recently suggested additional source of lower airway microbiota in CF patients is aspirated bile, which seems to affect the community composition significantly [60].

### Chronic obstructive pulmonary disease and smoking

Chronic obstructive pulmonary disease (COPD) is a progressive lung disease that is mainly defined by irreversible airflow limitation, small airway fibrosis, mucus hypersecretion, and tissue destruction [61–63]. The leading cause of COPD in the developed world is exposure to tobacco smoke, and in developing countries indoor air problems from biomass fuel combustion also increase the occurrence [64]. It is not known why some of the exposed develop the disease and others do not, but a microbial component in the etiology is possible, as the lung microbiome seems to differ between healthy and affected individuals [40, 47, 65–67].

Several studies suggest that COPD patients carry bacteria in their lungs that are not present in healthy individuals. These bacteria include for example *Moraxella*, *Curvibacter*, *Corynebacterium* [65], and dominance of *Pseudomonas* [42]. However, a general consensus about the significance of individual taxa and their involvement in the pathogenesis of COPD is still lacking. Attempts to define the association between COPD and microbial diversity in the lungs have given rather contradictory results: it has been suggested that the COPD lung microbiome is richer [67] and more diverse (Shannon and inverse Simpson) [47], that there is no difference in richness and evenness between healthy and affected lungs [10, 65], and that healthy lungs carry a more diverse microbiome (non-parametric Shannon) [42]. This inconclusiveness is probably linked to several factors: the interpersonal variation between lung microbiota is large and influenced heavily by the oral microbiome [47]; different lung compartments are dominated by different bacteria [42, 67]; and different levels of disease severity and medication affect the microbial community structure and diversity [47]. For instance, inhaled corticosteroids that are commonly used to treat COPD and asthma change the microbiome, advance enrichment of many community members, and may promote bacterial colonization in the airways [68]. In addition, the number of study subjects, sampling, selected laboratory protocols and analysis methods play a crucial role in the reliability of results.

Regarding smoking, in a recent thorough study comprised of sixty-four participants, the authors conclude that, although the data supports the existence of differences between the oral microbiome of “healthy” smokers and non-smokers, this does not seem to be the case regarding the lung communities, suggesting that the latter are not significantly altered by smoking [44]. An earlier study suggests that there is lower bacterial diversity (non-parametric Shannon) in smokers with moderate to severe COPD when compared to “healthy” smokers and non-smokers (the last two groups appearing to have no significant differences overall) [42]. Other studies have supported the hypothesis that lung bacterial communities don’t differ significantly between smokers and “healthy” smokers, while also adding to the evidence for differences in patients with severe COPD [45, 66].

### Asthma

Asthma is a complex, chronic disorder where inflammation-related changes in the airways lead to airflow limitation, causing recurring symptoms such as coughing, wheezing and breathlessness [69]. While the actual causes of asthma remain obscure, work conducted in recent years has unravelled a possible role for microbiota in either promotion of or protection from the disease [11, 70, 71]. As in other medical conditions discussed in this review, the data is strongly suggestive of a distinct pulmonary microbial community in healthy versus asthmatic people. Several studies suggest associations between environmental microbial exposure and the risk of developing asthma [72–75], or a role for antibiotic-induced gastrointestinal tract community effects during the first years of life [76–79]. The latter hypothesis is supported by the concept of a compartmentalized “common mucosal immune system”, in which microbe-induced differences in immunity at one mucosal site (e.g. gut) can affect immune function at other sites, including the mucosal surfaces of the airways [80–82].

Diverse studies, based on methods such as culturing, PCR, and/or DNA sequencing, have shown an association between asthma and certain microbial taxa. Implicated taxonomic groups include *Mycoplasma pneumoniae* and/or *Chlamydophila pneumoniae* [83–87]; *Moraxella catarrhalis*, *Haemophilus influenzae*, and/or *Streptococcus pneumoniae* [88, 89]; significantly more Proteobacteria, particularly *Haemophilus* spp., as well as *Staphylococcus* spp., but less Bacteroidetes, especially *Prevotella* spp. [40]; increased abundance of ~100 bacterial taxa distributed into 31 families, mostly Proteobacteria [90]; and higher levels of Proteobacteria, especially Gammaproteobacteria, in asthma cases [91]. Some studies report higher bacterial diversity in asthma [90, 91], while one study found no difference in diversity between controls and asthmatics [92]. Fungi have also been investigated in the context of asthma, with 90 fungal species showing higher relative abundance in sputum samples from asthma cases (particularly *Psathyrella candolleana, Malassezia pachydermatis, Termitomyces clypeatus* and *Grifola sordulenta*) while 46 were more abundant in controls (particularly *Eremothecium sinecaudum, Systenostrema alba, Cladosporium cladosporioides* and *Vanderwaltozyma polyspora*) [93]. *Malassezia pachydermatis,* which has been associated with atopic dermatitis as well as other atopic conditions, was present in asthma patients but not in the control group. Viral infections at early ages have also been associated with asthma [75].

Whether the aforementioned associations represent a causative or mediating role for microbiota in asthma, or reflect the result of community disturbances due to other causes is unclear [71]. A potential confounding factor that may affect conclusions derived from some of these studies is the use of patients that are under medical treatment (e.g. [40, 90]) which may produce anomalous microbial communities as a consequence. This is a potential problem for clinical studies in general, and extra care and consideration should be given to disentangle the effects of medication (e.g. antibiotics) from the real signal. A particular confounder in asthma studies could be the use of inhaled corticosteroids, which are immunosuppressive and could affect the lower airway microbiome. However, the results of a study where most of the asthmatic subjects were not using corticosteroids [91] were in line with older studies where corticosteroid use was not controlled for [40, 90]. See also [92, 94].

### Other conditions

It has been reported that the bacterial and fungal communities in the lower airways of lung transplant recipients differ from those of their upper airways, as well as from the upper and lower airways of healthy subjects [95, 96]. Although one small study suggests that the lower airways of transplant recipients have higher microbial alpha diversity (Shannon and inverse Simpson indices) than those of healthy subjects [96], most studies have concluded that the diversity is lower in transplant recipients’ airways (species richness and Shannon index) [12, 95, 97]. According to one study, the risk of bronchiolitis obliterans syndrome (BOS) is higher when the post-transplantation microbial community is distinct from the pre-transplantation state, while reestablishment of the pre-transplantation community has a protective effect, even when the subjects have CF and the community includes the potentially pathogenic *Pseudomonas* [97]. In a recent study, lung transplant recipients were found to have communities dominated by either *P. aeruginosa,* identified by both culturing and sequencing and associated with worse clinical status, or by *P. fluorescens,* which was only detectable by molecular methods, and typically found in asymptomatic subjects [12].

Since HIV patients have an increased risk of recurrent pneumonia, their lower airway microbiome has been studied, and there is a large multicenter research project, the Lung HIV Microbiome Project (LHMP), dedicated to it [44]. A high abundance of *Tropheryma whipplei* has been suggested as characteristic to HIV patients [52]. Antiretroviral therapy appears to have an effect on the microbiota [52, 98]. A metabolomics study using liquid chromatography-high-resolution mass spectrometry found that BAL samples from healthy subjects and HIV-1 infected patients had different metabolite profiles, and since many metabolites remained unidentified while one was identified as a siderophore of *P. aeruginosa,* the difference might be due to different microbiota [99]. While most of the airway microbiome publications cited in this review have studied Western populations, a microarray-based study compared BAL samples from HIV-infected pneumonia patients in Uganda [100] to an earlier study of a similar cohort from San Francisco [98], concluding that the Ugandan population had more diverse airway microbiota, and that several pathogenesis-related pathways were enriched in their predicted metagenome [100].

Several recent publications have addressed non-cystic fibrosis bronchiectasis. One study suggested that the complex microbial community present in the patients’ lower airways remains unchanged during exacerbations [101]. Another study found that community composition and diversity, particularly species richness, are correlated with lung function [102], leading to the proposal of a microbiome-based stratification system for predicting exacerbations, where patients with high relative abundance of *P. aeruginosa* or *Veillonella* are more likely to suffer frequent exacerbations than those with high abundance of *H. influenzae* [103].

Recently, an association between the lung microbiome, idiopathic pulmonary fibrosis (IPF) and disease progression has been identified [104, 105]. IPF is a progressive lung disease with unknown etiology that leads to respiratory failure and death typically within three to five years of diagnosis. It has been shown that patients with IPF have more than twofold higher pulmonary bacterial load compared to healthy individuals, and increased abundances of *Haemophilus*, *Neisseria*, and *Veillonella* [105]. Furthermore, the disease progression is associated with increased relative abundances of *Streptococcus* and *Staphylococcus*, and the overall bacterial burden: patients with highest bacterial load had more rapidly progressing disease, and a substantially increased risk of death [104, 105]. Further research is needed to determine if the changed microbiome is a cause or the result of IPF, but the findings provide support for testing antimicrobial therapy in IPF.

Other conditions that have been studied in relation to the microbiome of the lower airways so far include tuberculosis [106], interstitial lung disease [107], arsenic exposure [108] and intubation [6].

## Conclusion

The study of the human lower airway microbiome is still in its infancy. The research suggests that, contrary to what was previously thought, the lower airways are inhabited by a diverse bacterial community. Although the evidence is not conclusive, it has strong support from a variety of studies performed throughout the history of the field, using different and complementary methods. Regardless of what the true scenario is, there is little consensus on the actual impact of specific bacterial taxa, or of the community structure and diversity on human health and disease. At present, published studies suggest that healthy and affected (e.g. CF, COPD, asthma) lungs carry partly distinct bacterial communities. Still, it is not known whether this difference is a cause or a consequence of the disease, and whether the varying bacterial communities reflect the disease severity, or if it is actually the medication that alters the community structure. As for healthy lung communities, it is at present not possible to differentiate between a mutualistic role for bacteria in promoting health as opposed to an essentially neutral, purely commensal role. Additionally, the amounts of study subjects in published papers are relatively small, the used methods rather dissimilar, and the differences between studies also suggest broad interindividual variation. More controlled studies are needed to solve the role of the lower airway microbiome in health and disease. Based on current data, larger study cohorts, standardised sampling protocols and laboratory procedures to avoid any misinterpretation caused by contamination, as well as carefully selected sequencing and detection methods are essential in future projects.

## Abbreviations

BAL: Bronchoalveolar lavage
BOS: Bronchiolitis obliterans syndrome
CF: Cystic fibrosis
COPD: Chronic obstructive pulmonary disease
ILD: Interstitial lung disease
IPF: Idiopathic pulmonary fibrosis
NGS: Next generation sequencing
OTU: Operational taxonomic unit
PCR: Polymerase chain reaction
rRNA: Ribosomal ribonucleic acid
